# Highly Sensitive Colorimetric Biosensor for Staphylococcal Enterotoxin B by a Label-Free Aptamer and Gold Nanoparticles

**DOI:** 10.3389/fmicb.2018.00179

**Published:** 2018-02-13

**Authors:** Bhairab Mondal, Shylaja Ramlal, Padma S. Lavu, Bhavanashri N, Joseph Kingston

**Affiliations:** Microbiology Division, Defence Food Research Laboratory, Mysore, India

**Keywords:** gold nano particles, aptamer, enterotoxin B, colorimetry, biosensor

## Abstract

A simple, sensitive and selective colorimetric biosensor for the detection of Staphylococcal enterotoxin B (SEB) was developed using SEB-binding aptamer (SEB2) as recognition element and unmodified gold nanoparticles (AuNPs) as colorimetric probes. The assay is based on color change from red to purple due to conformational change of aptamer in the presence of SEB, and the phenomenon of salt-induced AuNPs aggregation which could be monitored by naked eye or UV–vis spectrometer. Results showed that the AuNPs can effectively differentiate the SEB induced conformational change of the aptamer in the presence of a given high salt concentration. A linear response in the range of 50 μg/mL to 0.5 ng/mL of SEB concentration was obtained. The assay was highly specific to SEB as compared to other related toxins. The limit of detection (LOD) of SEB achieved within few minutes was 50 ng/mL visually and spectrometric method improved it to 0.5 ng/mL. Robustness of the assay was tested in artificially spiked milk samples and cross-checked using in house developed sandwich ELISA (IgY as capturing and SEB specific monoclonal as revealing antibody) and PCR. This colorimetric assay could be a suitable alternative over existing methods during biological emergencies due to its simplicity, sensitive and cost effectiveness.

## Introduction

Development of highly sensitive, rapid response and super miniaturized sensors against toxin which can be weaponized to even a sub toxic dose is of uttermost importance for food security. Staphylococcal enterotoxin B (SEB) with its high stability to thermal and proteolytic activity is the primary cause of food poisoning and induces gastrointestinal symptoms such as emesis and diarrhea currently listed as a category B Bio-weapon agent (Singh et al., 2010). It is estimated that the lethal dose of SEB aerosols is 0.02 μg/kg and it can induce an emetic response at as low as 0.4 ng/kg in human (Balaban and Rasooly, 2000). Therefore, a rapid, sensitive, reliable and low cost detection of low concentrations SEB would be extremely desired.

Over the last few years, several sensitive and selective methods to detect SEB have been reported based on different detectors. These are based on electrical methods, electrochemical sensor (Chatrathi et al., 2007), magneto elastic sensor (Ruan et al., 2004), piezoelectric sensors (Lin and Tsai, 2003), cantilever sensor (Campbell et al., 2003) and electrical percolation-based sensor (Yang M et al., 2010). Meanwhile, a number of optical method have also been developed based on Surface Plasmon Resonance (SPR), and Chemiluminescence (Yang et al., 2009; Zhu et al., 2009) and apparatus-based detections such as LC, LC–MS (Sospedra et al., 2012). Most of the above referred methods utilizing antibodies as ligands molecule for binding and capture targets. Most of these methods used antibody as ligands and their high sensitivity and selectivity are coupled with sophisticated equipment, trained personnel and relatively long analysis time. Moreover, antibodies also have some restrictions as ligands in bioassays and biosensors. Antibody production is expensive and use animals for antibody production cause ethical problems. Additionally, antibodies are functional under specific environment and become unstable or inactive upon modification which make them unfavorable for the environmental or food samples analysis. Traditional immuno fluorescence assays employing fluorescent molecule have disadvantages due to their unstable optical and chemical properties and could be absorbed by biological samples to induce auto fluorescence and produced light scattering background (Wang and Liu, 2009). These limitations attract intense interest of researchers to find simple, cost-effective and comparatively fast new analytical methods with latest recognition elements for bio sensing applications.

Recently, aptamer became attractive candidates to replace antibodies due to several significant advantages in the field of diagnostics. Aptamers can be selected *in vitro* employing any target analyte and exhibited specific binding ability by changing conformation from random coil structures to rigid tertiary structures like hairpin or G-quadruplex. Moreover, small size, easy to handle, high target binding affinity, ease of synthesis, labeling, regeneration, lack of immunogenicity, inexpensive production make them attractive for pre-analytical sample processing and bio diagnostic assay development (Famulok and Mayer, 2011).

On the contrary, colorimetry is a notable technique used for routine food and clinical sample analysis. Gold nanoparticles (AuNPs) have emerged as a colorimetric indicator has become very attractive in colorimetric assays due to their simplicity, high extinction coefficients and strongly distance-dependent optical properties (Rosi and Mirkin, 2005). Hence, the development of aptamer-AuNPs colorimetric biosensors can be a simplified, attractive alternative detection system. Many researchers extended the target to various analytes, such as protein (Zhang et al., 2010), metal ion (Li et al., 2009; Wang et al., 2010) small molecule (Xu et al., 2009) and bacterial cells (Lavu et al., 2016) employing aptamers as recognition and AuNPs as a colorimetric indicator. However, we were unaware of any exclusive study discussing on aptamer-AuNPs based colorimetric assay for detection of SEB. In our previous report we have reported a single-stranded DNA aptamer (SEB2) that binds to enterotoxin B and able to detect in low nanomolar range (Mondal et al., 2015).

In this work, we have the demonstration of colorimetric detection of SEB using AuNPs as indicator and aptamer as specific recognition probe. SEB was detected by monitoring the color change of the AuNPs with naked eye as well as spectrometrically. We have optimized several assay parameters and performance of the assay. The effectiveness of developed assay was evaluated using artificially spiked milk and naturally contaminated samples. Altogether, this method is simple, rapid, and highly sensitive and extends the available detection methods for SEB during biological emergencies.

## Materials and methods

### Reagents and chemicals

Luria–Bertani (LB) broth, LB agar, Baird–Parker agar base, and egg yolk (EY) tellurite enrichment, Tryptic Soy Broth (TSB) and Brain heart infusion (BHI) broth were obtain from Himedia (India). Potassium phosphate dibasic, citrate protected gold nano particle (20 nm), glycine, sodium chloride (NaCl), sodium hydroxide, o-Phenylenediamine dihydrochloride (OPD), Dulbecco's phosphate buffer saline (DPBS), bovine serum albumin (BSA), and all PCR reagents obtained from Sigma-Aldrich (India).

Synthesized single-stranded DNA (ssDNA) aptamer (SEB2) 5′TAGCTCACTCATTAGGCACGGGTAGGCCATAATATCTTATTAGCGTAATTCTGCGATTGGCATAGTTAAGCCAGCC3′ (Mondal et al., 2015) and random ssDNA (RDNA) 5′CGTAGTCTAGTGTCGATTAGTTTCCTTGAGACCTTGTGCT3′ were obtained from Xcelris Bioscience (Ahmadabad). DNA stock solution was prepared in 10 mM DPBS (pH 7.0) and was stored at 4°C before use. All other reagents were of analytical reagent grade and ultra-pure water (Milli-Q plus, Millipore Inc) used throughout the experiments.

### Bacterial strains and culture

The bacterial reference strains used in the study are listed in Table 1. Staphylococcal and non-staphylococcal cultures were grown in Brain heart infusion (BHI) broth under aeration at 37°C for 18–24 h. *S. aureus* cells were grown in Tryptic Soy Broth (TSB) for 24 h for enterotoxin production. Cultures were cryopreserved in 15% glycerol and stored at −80°C.

**Table 1:** List of bacterial strains and r-protein used in the study.

**Sl. No**.	**Bacterial strains**	**Source**	**ELISA**	**PCR**	**Colorimetric biosensor**
1.	*Staphylococcus aureus*	FRI 722	+	+	+
2.	*Staphylococcus aureus*	ATCC 29213	+	+	+
3.	*Staphylococcus aureus*	NCIM 2122	+	+	+
4.	*Staphylococcus aureus*	NCIM 2120	+	+	+
5.	*Staphylococcus aureus*	NCIM 2127	−	−	+
6.	*Bacillus cereus*	ATCC 10876	−	−	−
7.	*Burkholderia pseudomallei*	NCTC 10274	−	−	−
8.	*Clostridium perfringens*	Isolate	−	−	−
9.	*E. coli*	ATCC 10536	−	−	−
10.	*Enterococcus* spp.	Isolate	−	−	−
11.	*Klebsiella pneumoniae*	ATCC 13883	−	−	−
12.	*Lactobacillus* spp.	Isolate	−	−	−
13.	*Listeria monocytogenes*	ATCC 19114	−	−	−
14.	*Proteus vulgaris*	ATCC 33420	−	−	−
15.	*Pseudomonas* spp.	Isolate	−	−	−
16.	*Salmonella paratyphi A*	ATCC 9150	−	−	−
17.	*Shigella flexneri*	ATCC 9199	−	−	−
18.	*Streptococcus* spp.	Isolate	−	−	−
19.	*Yersinia enterocolitica*	ATCC 23715	−	−	−
20.	*Vibrio parahaemolyticus*	ATCC 17802	−	−	−
21.	r-SEB protein	r-clone	+	+	+
22.	r-SEA protein	r-clone	−	−	−
23.	r-SEC protein	r-clone	−	−	−
24.	r-SEG protein	r-clone	−	−	−

### Instrumentation

The UV-vis absorption spectra and kinetics parameter was monitored using UV-vis spectrophotometer (TECAN, India).

### Detection of SEB using colorimetric bio sensing method

First, 50 μL of single-stranded SEB aptamer or random DNA (240 nM) and different concentrations (0.5 ng−50 μg/mL) of SEB in 10 mM PBS were added in 96-well microtiter plates for 30 min. Then, 50 μL of AuNPs solution was added and incubated for 5 min. Subsequently, 50 μL of 300 mM NaCl was mixed with the solution to provide a final volume of 150 μL. After the solution was equilibrated for 15 min, UV–vis absorbance was recorded at 520 nm. The UV–vis absorption measurement was carried out at the range from 400 to 850 nm. To developed colorimetric biosensing assay conditions such as, aptamer concentration, NaCl concentration, binding temperature, and binding time were investigated in this study. For evaluation of cross-reactivity among different related toxins and other proteins, the assays were repeated using r-SEA, r-SEC, r-SEG, and BSA.

### Specificity of the colorimetric biosensor

To evaluate the specificity of established colorimetric biosensing, toxins extracted from various staphylococcal and non-staphylococcal cultures mentioned in Table 1 were tested. Briefly, the bacterial cultures were grown in TSB medium for 24 h. Enterotoxins were extracted from samples by TCA precipitation method described by Vernozy-Rozand and co-workers (Nguyen, 2014). Extracted toxin was tested directly through colorimetric biosensing as mentioned earlier. For comparison, staphylococcal isolates were subjected to PCR analysis to examine the presence of *seb* gene using *seb* specific primers. In order to appraise the feasibility of the assay for direct detection in food matrix, r-SEB was spiked into milk (0.5 ng−50 μg/mL) and colorimetric biosensing was performed as mentioned earlier. Toxins from milk samples were extracted by TCA precipitation.

### Analysis on natural samples

To access the real time applications of colorimetric biosensing method, different natural samples were tested (Table 1) for detection of presence of enterotoxin B producing *S. aureus* through newly developed method. Enterotoxins were extracted from samples by TCA precipitation and subjected for detection in standardized assay condition.

### Comparison of colorimetric biosensor with available kits

The sensitivity and feasibility of the newly described method was evaluated by comparing with in-house developed sandwich ELISA and PCR kits. Toxin was extracted from various *S. aureus* culture broth and the colorimetric assay was performed. The UV–vis absorption of the colorimetric reactions was measured and the results were interpreted and compared with in house developed kit.

In house, sandwich ELISA was standardized using anti SEB chicken IgY(Anti SEB IgY) as capture antibody and murine monoclonal anti SEB antibody (SEB mAb) as revealing antibody followed by detection using HRP conjugated goat anti mouse IgG. Briefly, anti-SEB IgY was diluted to 1:500 in carbonate–bicarbonate buffer and coated in maxisorb 96-well plate (Nunc- Denmark) and incubated at 4 °C for overnight. After blocking with 250 μL of 1% BSA for 1 h, samples (culture supernatants/enriched samples/extracted samples) was added to the wells and incubated at room temperature (RT) for 45 min. The plate was incubated with secondary conjugate for 40 min and chromogenic reaction was carried out using o-Phenylenediamine dihydrochloride (OPD) and 30% H_2_O_2_ in citrate phosphate buffer. After each step, plate was washed thoroughly with PBST (Tween 20–0.05%) solution. Results were interpreted either by visual inspection or by measuring the absorbance at 450 nm in ELISA reader (TECAN, USA).

For PCR based detection, DNA was extracted from pure culture and PCR was performed in a 50 μl reaction mixture containing 1X of PCR buffer, 1.5 mM of MgCl_2_, 0. 20 mM of dNTPs, 1.25 U of *Taq* DNA polymerase and 5 pmol of each *seb* specific primer (Table S1).

## Results

### Principle of the colorimetric detection

**Figure 1:**
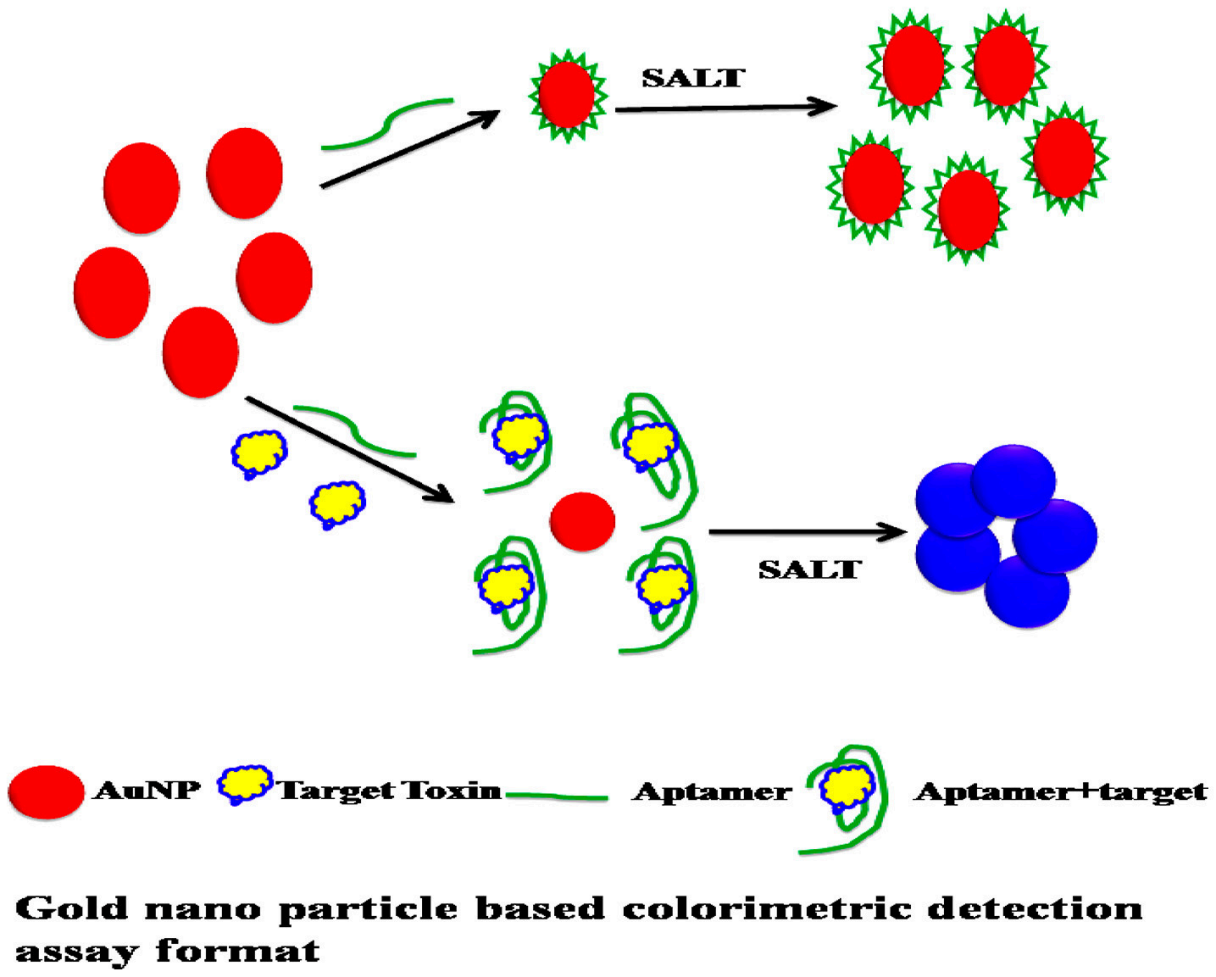
Gold nano particle based colorimetric detection assay format.

In this work, synthesized citrate protected AuNP solution was used to prevent the strong van der Waals attraction between AuNPs causing them to aggregate (Wang et al., 2010). The detailed principle was described in Supporting Information 1 (Figure 1).

**Figure 2:**
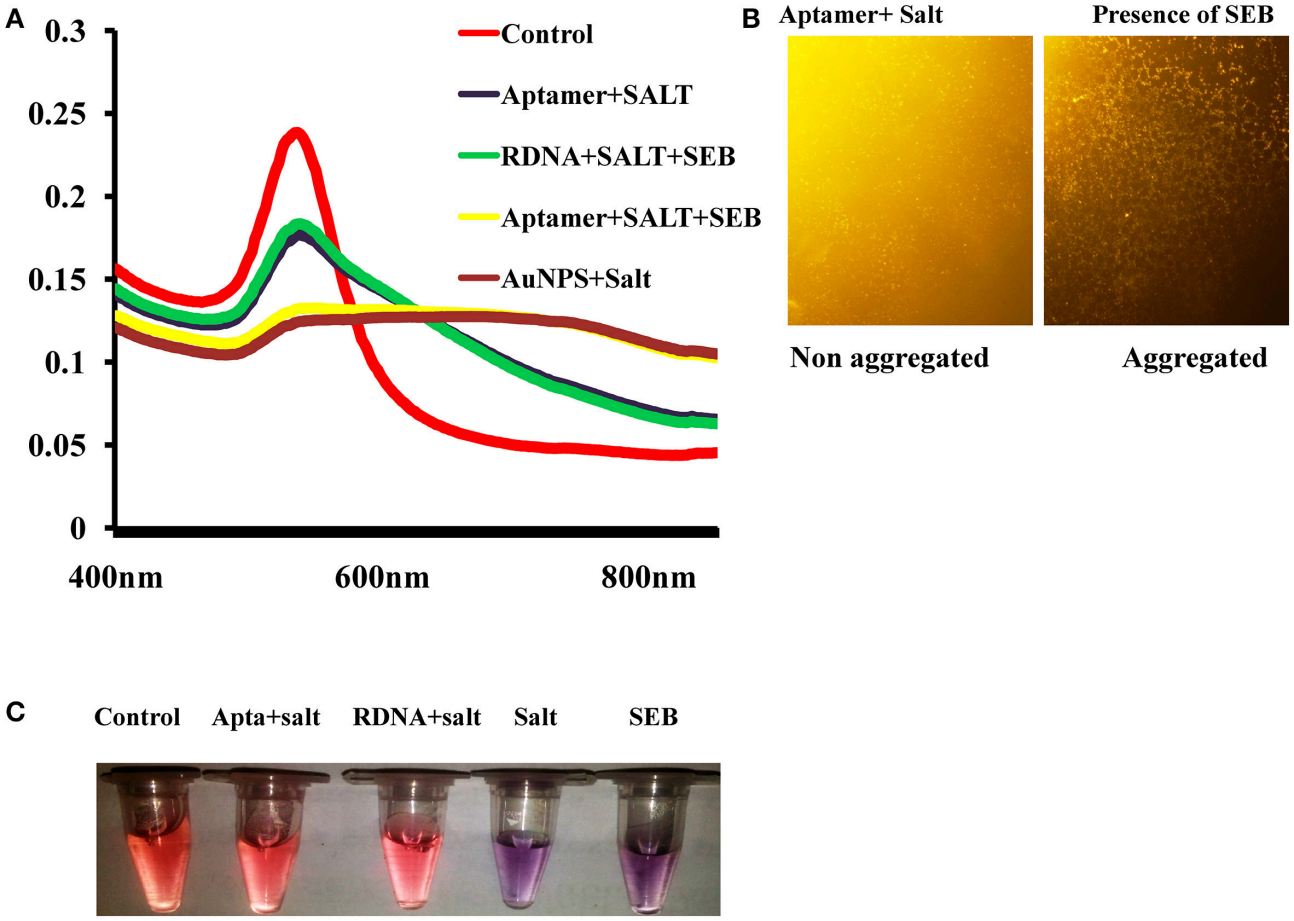
Spectral characteristic and visual observation of AuNPs solutions in various conditions. **(A)** Absorption spectra of AuNPs solutions in various conditions. **(B)** Microscopic image of AuNPs/aptamer mixed solutions in presence and absence of toxin. **(C)** Visual observation of AuNPs solutions in various conditions.

As we can see, the aptamer with a random coil structure could bind to AuNPs. After adding a high concentration of NaCl, AuNPs would remain dispersed. Upon the addition of target, SEB–binding aptamer (SEB2) induced the conformation to a rigid stem–loop structure. As a result, aptamer lost the ability to protect the AuNPs under high-salt conditions. Hence AuNPs aggregates and the color of AuNPs solution changes from red to purple (Figures 2A,B).

### Optimization of assay condition

Aggregated AuNPs tend to precipitate easily, so it is important to monitor the salt-induced AuNPs aggregation kinetics for colorimetric detection of SEB. To optimize, the performance of the developed assay various conditions such as, aptamer concentration, NaCl concentration, binding temperature, and binding time were investigated in this study.

To optimize the NaCl concentration in the absence or presence of SEB, various range of NaCl (0–500 mM) was added in predetermined volume of AuNPs solutions (Figure S1). The net absorbance of AuNPs solution reached the maximum at 300 mM NaCl at 620 nm. On the contrary, in the absence of SEB, the A620 nm reached a relatively high value at 350 mM NaCl and thus increased the interference from the background. Then, on basis of higher sensitivity and lower background signal, 250 mM NaCl was considered as suitable concentration for AuNPs aggregation.

**Figure 3:**
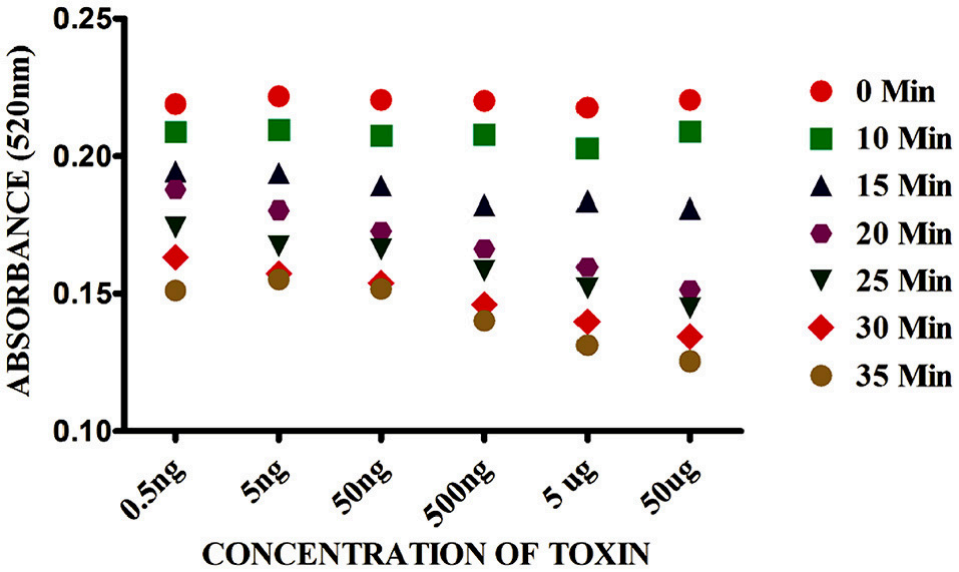
Effect of time on Gold nano particle based colorimetric detection assay. The absorption value profiles at 520 nm of the sensor for different concentrations of SEB.

The kinetic parameters of AuNPs aggregation was measured between 0 and 35 min in presence of SEB. As we can see in Figure 3 that the absorbance values of AuNPs at 520 nm decreased with the reaction time and reached maximum at 20 min and reached a constant. After 20 min, the absorbance increased very slowly. Considering the assay speed, 15 min were preferred as the assay time on the basis of higher sensitivity and time saving.

The effect of aptamer concentration also monitored for colorimetric detection of SEB. High concentration of aptamer is not suitable for detection of small amounts of the target because it can produce large background signal. The effect of the aptamer concentration was studied in the range of 0–350 nM (Figure S2). The optimum result was obtained at 240 nM aptamer concentration without any background, hence considered as suitable concentration for the detection of SEB.

The kinetic parameters of AuNPs aggregation in various temperatures (10–60°C) were monitored. Higher response was observed within the range of 10–30°C, and the response decreased at 30°C, this could be due to loss of secondary structure of aptamer at high temperature. Taking consideration of aptamer selection condition and most probable secondary (Figure S3) structure (Mondal et al., 2015), room temperature (27°C) was chosen as the suitable temperature for all experiments.

### Spectral characteristics of the colorimetric biosensor

UV–vis spectra of the AuNPs solution for colorimetric detection of SEB under different experimental conditions were shown in Figure 2A. As can be observed, after addition of salt to the AuNPs solution the surface Plasmon resonance (SPR) absorption band shifted and a new peak appeared at about 620 nm. Correspondingly, the AuNPs solution color also changed from red to purple. This shifting and color change was mainly due to salt-induced aggregation of AuNPs (Figure 2B). Only after the addition of SEB2 aptamer or random DNA a similar characteristic SPR absorption band of AuNPs at 520 nm appeared. We consider the surface of AuNPs was absorbed by ssDNA to protect the AuNPs from salt-induced aggregation (Lavu et al., 2016). However, after addition of SEB, a SPR band at about 620 nm was clearly observed and the color of the AuNPs solution changed from red to purple (Figure 2C).

To support the analysis, control experiments were performed using random DNA (RDNA) under the standardized condition. As could be observed, after the addition of SEB, there was not any change in UV–vis spectrum shift and the color of the AuNPs solution remained red. This indicates that SEB could binds to its aptamer, not to random DNA.

### Sensitivity of the colorimetric biosensor

**Figure 4:**
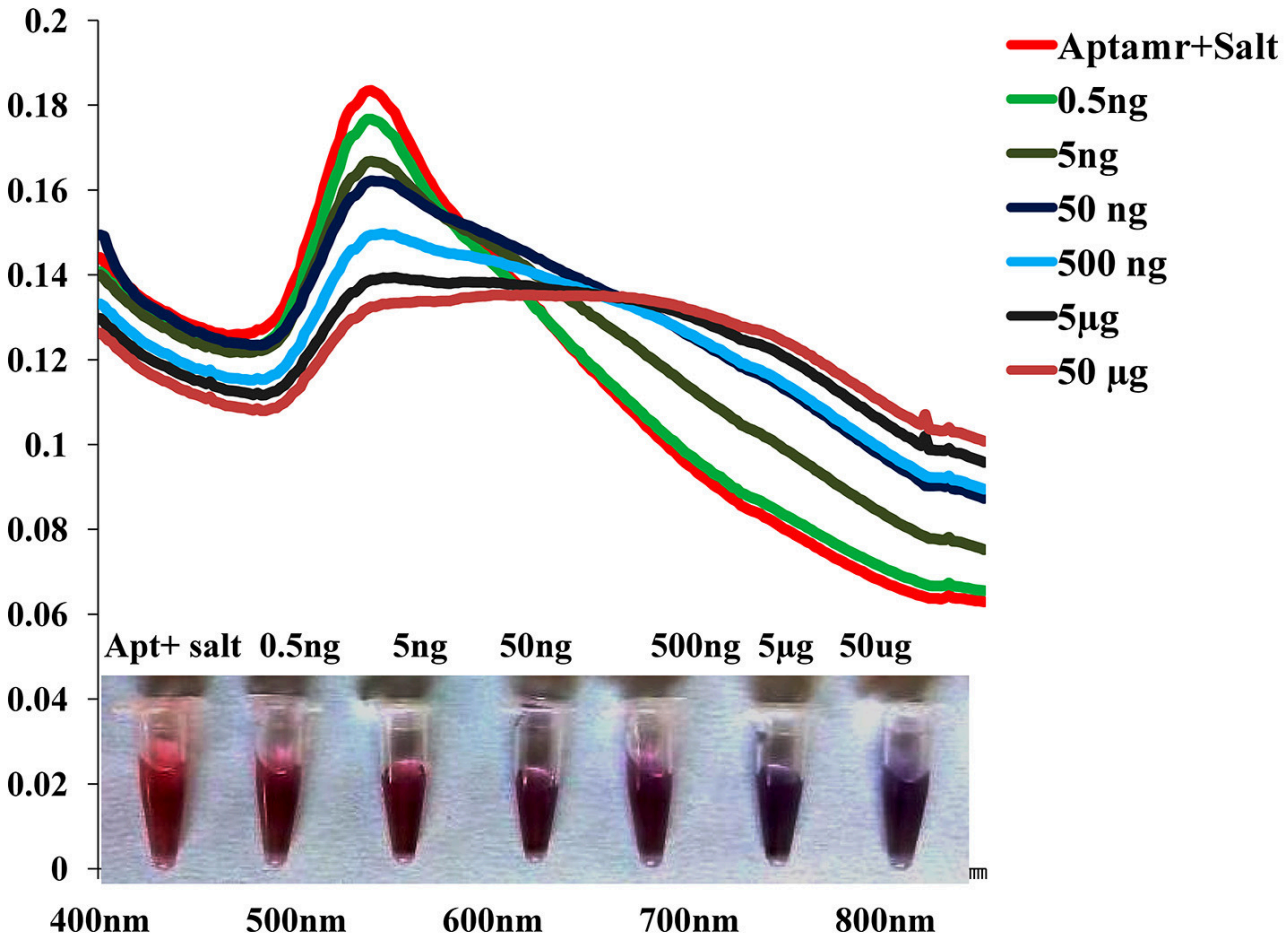
Sensitivity of Gold nano particle based colorimetric detection assay. Absorption spectra of AuNPs/aptamer mixed solutions in the presence of various concentration of SEB.

**Figure 5:**
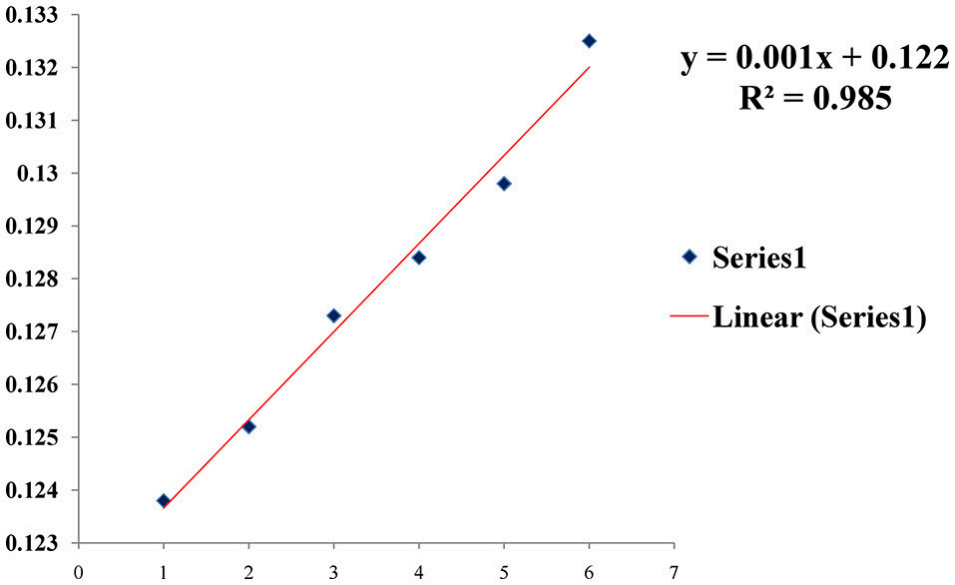
A linear correlation in presence of various concentration of toxin.

To monitor the sensitivity of colorimetric Apta sensor, the UV–vis spectra of AuNPs with different concentration of SEB in the range of (0–50 μg/mL) was monitored in the optimized experimental conditions (Figure 4). As can be seen in Figure 4, spectrometrically the limit of detection (LOD) was 0.5 ng/mL of SEB and even with the naked eye; we could identify 50 ng/mL of SEB within few minutes. Moreover, a linear correlation was obtained between A620 nm and the concentration from 0 to 50 μg/mL (Figure 5). This linear range and LOD could be comparable to the available detection system for SEB.

### Specificity of assay of the colorimetric biosensor

**Figure 6:**
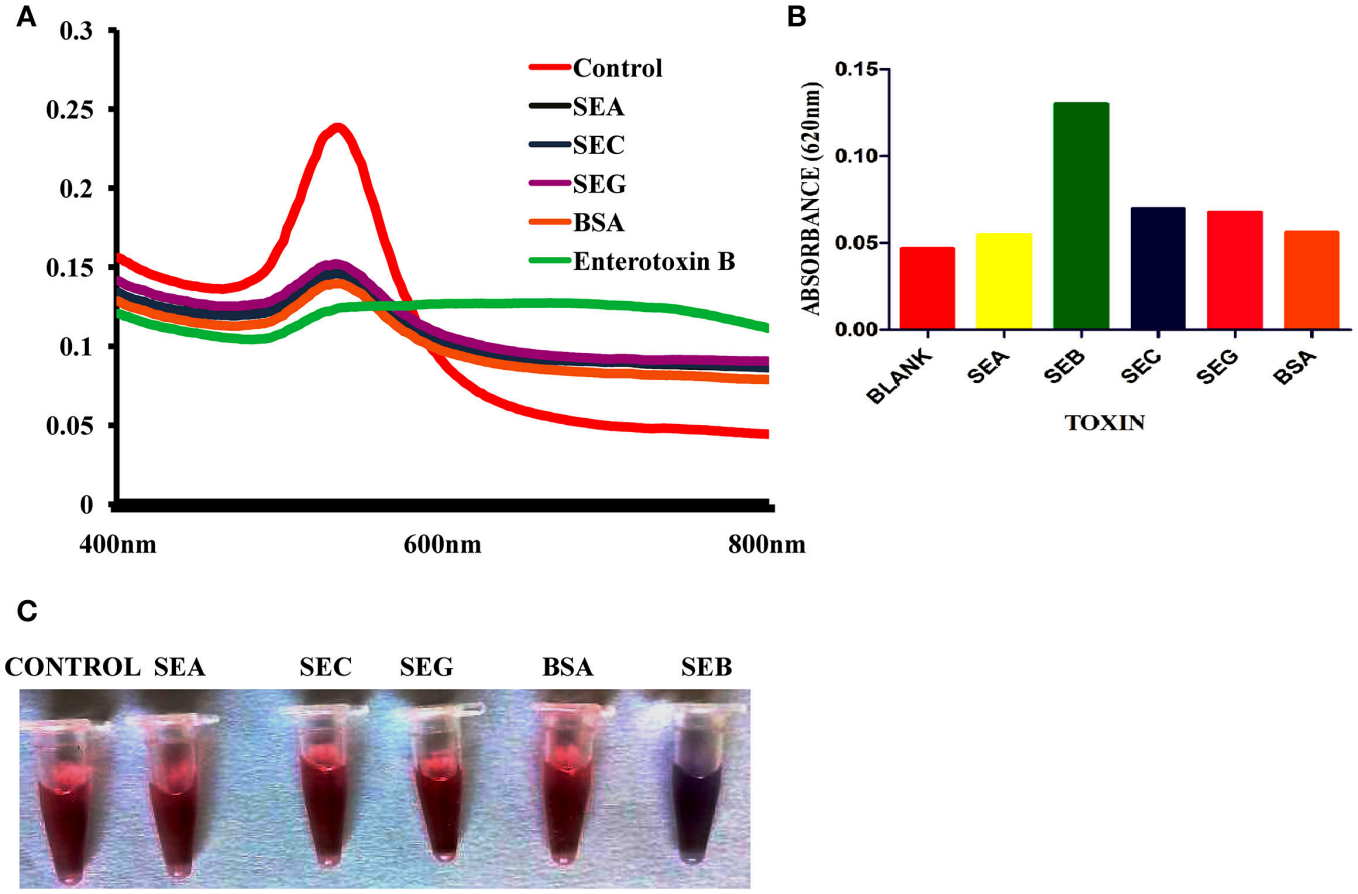
Selectivity of Gold nano particle based colorimetric detection assay. **(A)** Absorption spectra of AuNPs/aptamer mixed solutions in the presence of other related toxin. **(B)** Absorption value at 620 nm of AuNPs/aptamer mixed solutions in the presence of other related toxin. **(C)** Visual observation of AuNPs/aptamer mixed solutions in presence of other related toxin.

Selectivity of colorimetric biosensor was evaluated by testing with other related toxins and various bacterial cultures toxin extracts. Reactivity was observed only in SEB producing *S. aureus* strains and presence of r-SEB toxin. Result shows that there was no major change in the SPR band of AuNPs before and after the addition of other related toxins (Figures 6A–C) and other non-staphylococcal bacterial cultures protein extract (Table 1). Correspondingly, the red color remained unchanged. We inferred that the other related proteins had no specific binding to the SEB aptamer, thus, no aggregation occurred. However, in presence of SEB, AuNPs aggregates and the absorbance at 520 nm decreased and a SPR band at about 620 nm was clearly observed.

### Analysis of complex food and comparison with available kits

**Figure 7:**
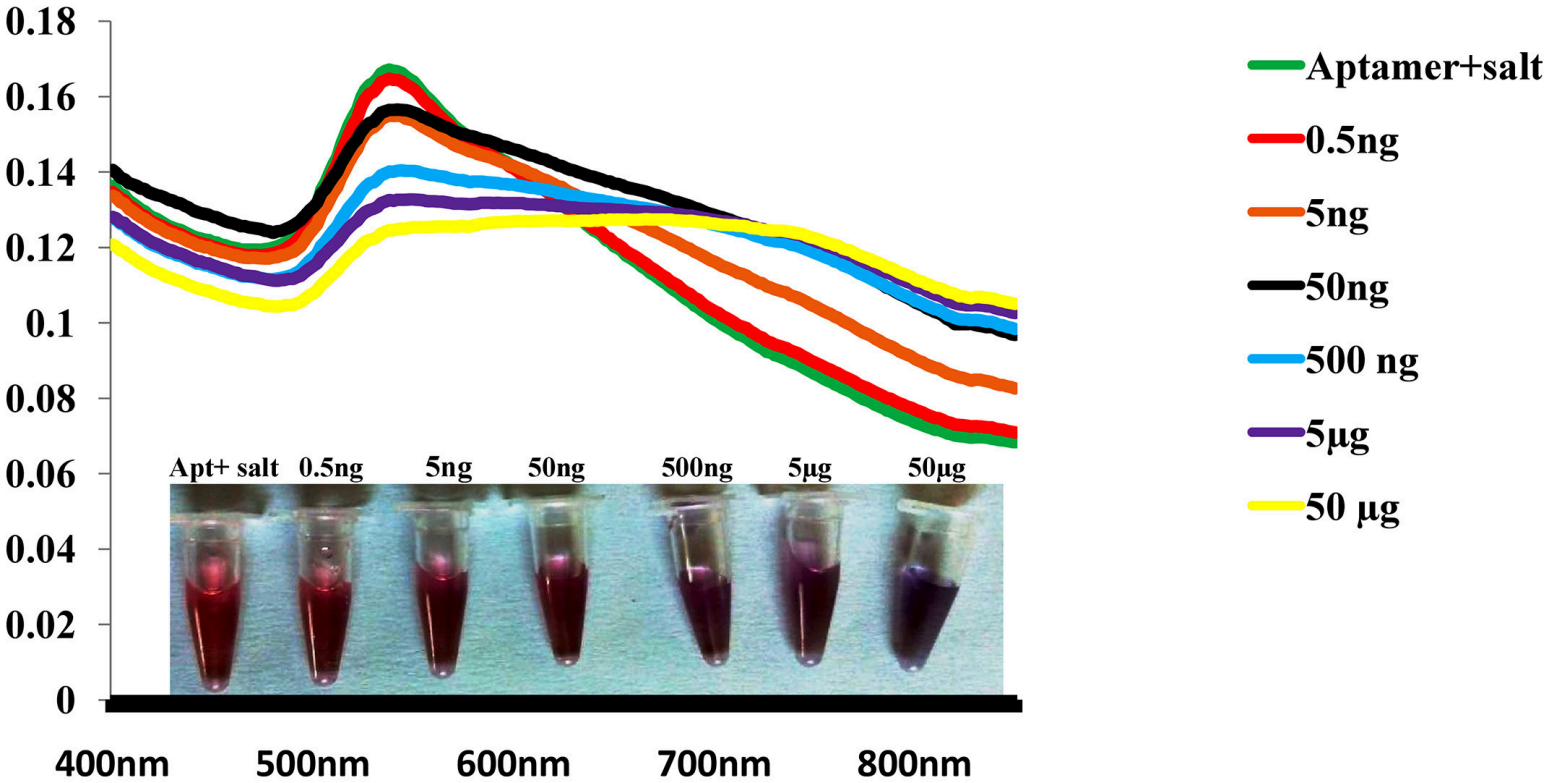
Robustness of Gold nano particle based colorimetric SEB detection assay. Absorption spectra of AuNPs/aptamer mixed solutions in spiked milk sample with various concentration of SEB.

Most common root of Enterotoxigenic *S. aureus* contamination is milk and milk based products, hence spiking study was performed in milk. The gold nano particle based colorimetric biosensor could detect r-SEB at level of 0.5 ng/mL without any hindrance in sensitivity (Figure 7). However, insignificant lower signal and absorbance values were observed in toxin extracted from spiked milk in comparison with actual recombinant toxins.This limitation can be ruled out by using a blank with similar composition of the sample.

Gold nano particle based colorimetric biosensor was assessed for its efficiency and practicability for detection of targets from natural food samples. Among 50 naturally contaminated food samples, the assay able to detect 15 positive sample for the presence of SEB. As shown in Table 2, the assay can able to detect SEB positive *S. aureus* from natural samples within 45 min. The comparison of result with in-house ELISA and PCR kit validate the same. Altogether,these experimental results demonstrated that this colorimetric biosensor based detection of SEB can be can be a suitable alternative tool for routine investigation of food and natural sample in laboratory during biological emergencies.

**Table 2:** Analysis of natural samples for enterotoxin B through Colorimetric biosensor.

**Sample type**	**No. of samples SEB positive for**		
	**Colorimetric biosensor**	**ELISA**	**PCR**
Milk (*n* = 10)	4	4	4
Cheese (*n* = 10)	3	3	3
Ice cream (*n* = 10)	3	3	3
Chicken (*n* = 10)	2	2	2
Pastries (*n* = 10)	3	3	3
Total (*n* = 50)	15	15	15

## Discussion

*Staphylococcus aureus* is a unique and notorious pathogen due to its wide range of virulence factor such as α -hemolysin, several enterotoxins (as many as 23, ranging from SEA to SHV), TSST and other secretary proteins (Balaban and Rasooly, 2000; Singh et al., 2010). Among the virulence, the heat-stable staphylococcal enterotoxins B (SEB) are the most effective cause of food-borne illness, as it cause infection nanogram levels, can cross gastro-intestinal barrier and easy to aerosolize which implies to use as potent biological weapon (Balaban and Rasooly, 2000; Mudili et al., 2015). Moreover, heat stable SEB can remain unaffected in food after cooking and even heat treatment which only inactivates or kills *S. aureus* cells. Hence, detection and quantification of SEB levels from food is a more accurate approach than quantifying total *S. aureus* viable counts (Reddy et al., 2014). Therefore, a simple and rapid method for screening SEB toxin would provide an important tool to prevent food poisoning. In this present study colorimetric detection strategy for detection of SEB was devised employing AuNPs as an optical indication and new generation affinity molecule aptamer as aptamer as specific recognition probe as an alternative to the traditional detection approach. The detection criterion is based on for color change (red to purple) due to aggregation of AuNPs (Wang et al., 2010; Lavu et al., 2016).

A large number of literatures are available in PubMed related detection of SEB from various substances (food, clinical and suspected samples) upon searching the keywords Enterotoxin B and detection for Advantages of the current method are as discussed below.

In our study we have employed new generation affinity molecule aptamer as recognition probe which is advantageous in comparison to available ligands in term of specificity, sensitivity and stability (Mondal et al., 2015; Lavu et al., 2016). Moreover it is known fact mammalian antibodies have affinity toward Protein A (SpA) which always leads to non-specific and false positive results limits their application of immunoassays (Reddy et al., 2014; Mudili et al., 2015). Conventional nucleic acid based need additional steps of sample purification and extraction of DNA and the sensitivity will be easily compromised by interfering matrix associated inhibitory substances (Mudili et al., 2015). In our study SEB specific aptamer as ligands overcome the existing limitation, without affecting the sensitivity. Specificity was also not hindered by other non-target bacterial species as well as SEB negative *S. aureus* strains. Thus, aptamer- AuNPs based method based assay developed in this study is most reliable.

In this research we employed unmodified gold nanoparticles (AuNPs) as colorimetric probes and labeled free aptamer as recognitions elements which offers better results compared to available method (thiolated and biotinylated ligands based colorimetric assay) in which modification of aptamer might lead to lose its affinity simultaneously affect the time and cost of assay (Stoltenburg et al., 2007).

Another advantage of the study, our assay based on color change the presence of SEB due to salt-induced AuNPs aggregation which could be monitored by naked eye or UV–vis spectrometer. The sensitivity and selectivity of the developed bioassays when investigated resulted in limit of detection (LOD) achieved within few minutes was 50 ng/mL visually and spectrometric method improved it to 0.5 ng/mL. Importantly, the detection system could detect SEB with high specificity and required neither the extraction of specific genes nor the precise probe or antibody (Yang et al., 2009; Zhu et al., 2009). Hence our assay is comparable with other available method where high sensitivity and selectivity are coupled with sophisticated equipment, trained personnel and relatively long analysis time. It is therefore better than most of current existing methods. Further, spiking studies exhibited the ability of the assay to detect SEB in presence of complex food matrix. The obtained result proved assay applicability for rapid detection from food samples, particularly on milk. Moreover, the limit of detection for the assays in terms of rapidity, sensitivity and cost are comparable to available antibody and molecular based methods (Campbell et al., 2003; Lin and Tsai, 2003; Ruan et al., 2004; Chatrathi et al., 2007; Yang et al., 2009; Zhu et al., 2009; Yang M et al., 2010; Sospedra et al., 2012).

Altogether, this described strategy having potential for development of rapid and sensitive multiplex detection system for common enterotoxin in both in field and laboratory based routine analysis over a wide range of food, clinical and environmental samples. Our approach is convenient to achieve rapid, semi quantitative detection via visual inspection or quantitative detection via visible light absorbance spectroscopy. Taken together, these advantages make this technology an appealing choice for future development of point-of-care testing.

## Conclusion

The paper introduced a label free biosensor to detect SEB using SEB binding aptamer ligands and AuNPs as indicator. Presented biosensor has several advantages compared to available biosensor for SEB detection. First, the biosensor based on color change of AuNPs solution from red to purple in presence of SEB, which can be seen through naked eye as well as spectrophotrometrically. Second, the detection limit of the biosensor is as low as 0.5 ng/mL and the entire assay can be completed in less than 45 min. Third, the developed sensor does not require sophisticated instrument and it uses minimum reagents hence reduce the cost of SEB detection compared with available detection method for SEB.

## Author contributions

BM: Writing, performing experiment, Experiment design, Data interpretation, result analysis. SR: Writing, Experiment design, Data interpretation. PL: Writing, Experiment design, Data interpretation, result analysis. BN: Performing experiment, Data interpretation, result analysis. JK: Writing, Experiment design, Data interpretation.

### Conflict of interest statement

The authors declare that the research was conducted in the absence of any commercial or financial relationships that could be construed as a potential conflict of interest.
