# Supplementary material for: Highly Sensitive Colorimetric Biosensor for Staphylococcal Enterotoxin B by a Label-Free Aptamer and Gold Nanoparticles
Source: Front Microbiol. 2018 Feb 13;9:179. doi: 10.3389/fmicb.2018.00179 (PMC5816949; doi:10.3389/fmicb.2018.00179)

**Supporting Information**

**Category of paper:**

**Original Article**

**Article Title:**

**Highly Sensitive Colorimetric biosensor for Staphylococcal enterotoxin B by a Label-Free Aptamer and Gold Nanoparticles**

**Authors:**

Bhairab Mondal, Shylaja Ramlal*, Bhavnashri.N, Padma Sudharani Lavu, and Joseph Kingston.

**Institution:**

Defence Food Research Laboratory

Address:

Microbiology Division, Defence Food Research Laboratory, Siddarthanagar, Mysore, Karnataka,

India -570011.

*** Corresponding author address:**

Dr. Shylaja R, Sc D,

Defence Food Research Laboratory,

Siddarthanagar,

Mysore, Karnataka,

India -570011.

Email: **shylaja@dfrl.drdo.in**

Phone No. +91 9480447388

**Supporting Information.1**

**Principle of the colorimetric detection**

A high concentration of salt solution would neutralize the negative charge of citrate and lead to the AuNP aggregation and the color changes from red to purple (15, 20). However, it has been reported ssDNA (SEB2 aptamer) with a random coil structure could uncoil and adsorbed onto the surface of AuNPs through the coordination interaction between the nitrogen atoms of the exposed bases and the AuNPs (15, 20). Thus, DNA phosphate backbone with negative charges masked the surface of AuNPs. The electrostatic repulsion prevented the strong van der Waals attraction and enhanced the stability of AuNPs against salt-induced aggregation and retains their red color under high salt conditions (20). Based on this rationale, an aptamer-based colorimetric biosensing strategy can be well developed for the detection of Enterotoxin B.

| **Primer** | **Oligonucleotide Sequence (5′-3′)** | **Amplicon size (bp)** |
| --- | --- | --- |
| Normal *seb* | F – GAGAGTCAACCAGATCCTAA  R – CTTTTTCTTTGTCGTAAGAT | 719 |

**Supporting Table 1: Primers used for PCR amplification**

**Supporting Figures:**

**Supporting fig.S1. The effect of the concentration of NaCl**


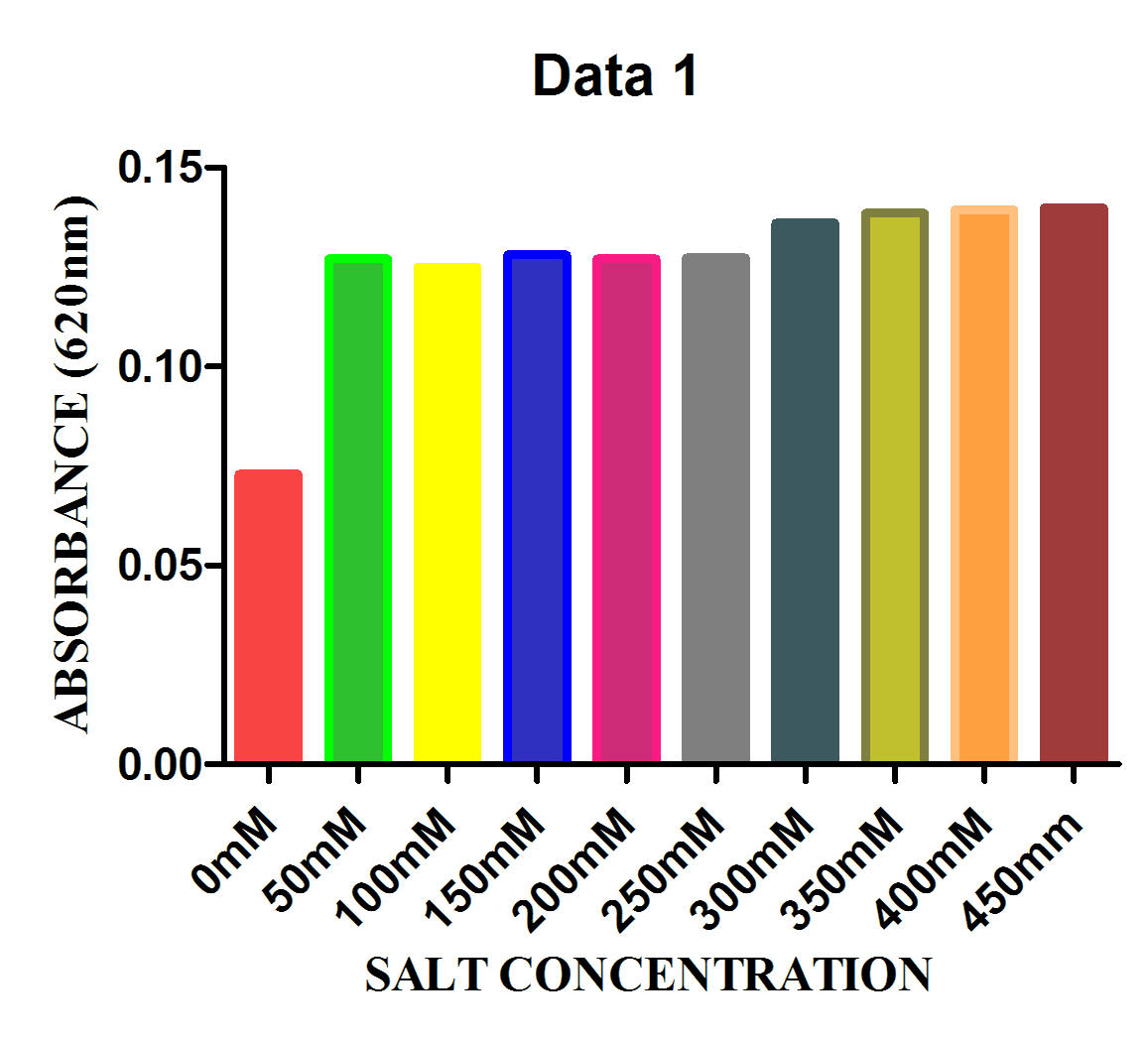


**Supporting fig.S2. The effect of the aptamer concentration**

**Supporting fig.S3. Possible Secondary structure by Mfold software**


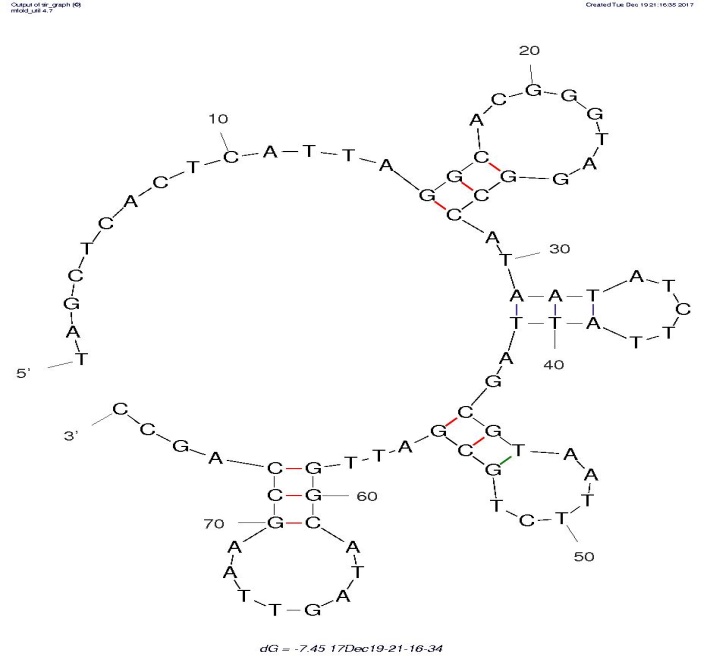

Supplement: Supplementary file 1 [file Table1.DOCX]
